# Efficacy and safety of low-dose thrombolysis in acute intermediate-high risk pulmonary thromboembolism complicated by PaO_2_/FiO_2_ < 300

**DOI:** 10.3389/fmed.2026.1849541

**Published:** 2026-07-09

**Authors:** Lei Liu, Congcong Li, Liang Shi, Debin Ma, Zhiyuan Zhang, Hongzhu Bao, Chunhua Li, Li Li, Min Wang, Zhuang Ma, Junli Zhang

**Affiliations:** Department of Respiratory and Critical Care Medicine, General Hospital of Northern Theatre Command, Shenyang, China

**Keywords:** alteplase, clinical efficacy, intermediate- high risk, low-dose thrombolysis, PaO2/FIO2 ratio, pulmonary thromboembolism, safety

## Abstract

**Objective:**

This study aimed to evaluate the clinical efficacy and safety of low-dose alteplase thrombolysis in patients with acute intermediate-high-risk pulmonary thromboembolism (PTE) and PaO_2_/FiO_2_ ratio < 300 mmHg.

**Methods:**

A total of 96 patients with acute intermediate-high-risk PTE and PaO_2_/FiO_2_ ratio <300 mmHg were enrolled between October 2020 and February 2025. Patients were randomly assigned to conventional-dose thrombolysis (alteplase 50 mg/2 h), low-dose thrombolysis (alteplase 25 mg/2 h), or low-molecular-weight heparin anticoagulation. PaO_2_/FiO_2_ ratio and NT-proBNP were assessed at baseline, 24 h, 3 days, and 7 days, while mPAP was measured at baseline, 24 h, and 7 days. Safety endpoints included mortality, hemodynamic decompensation, and bleeding within 7 days. Secondary outcomes included hospital and ICU stay, costs, and 3-month outcomes.

**Results:**

Baseline characteristics were comparable among the three groups. Both thrombolysis groups showed greater improvements in PaO_2_/FiO_2_ ratio at 24 h, 3 days, and 7 days compared with the LMWH group (*p* < 0.05), with no difference between thrombolysis regimens. NT-proBNP levels decreased more in the thrombolysis groups at 24 h and 3 days (*p* < 0.05), with no difference at day 7. Similarly, mPAP was significantly lower in the thrombolysis groups at 24 h and 7 days (*p* < 0.05), without intergroup differences. No major bleeding occurred. Minor bleeding was lower in the low-dose group than in the conventional-dose group (6.1% vs. 20.5%, *p* < 0.05). No differences were observed in hospital stay, ICU stay, costs, symptom improvement, or chronic thromboembolic pulmonary disease at 3 months (all *p* > 0.05).

**Conclusion:**

In patients with acute intermediate-high-risk PTE and a PaO_2_/FiO_2_ ratio <300 mmHg, low-dose thrombolysis yielded early improvements in oxygenation, mPAP and right ventricular function, with efficacy similar to conventional-dose thrombolysis. We found a numerical reduction in minor bleeding events and no major bleeding across groups. However, the study lacked sufficient statistical power to confirm a true difference in bleeding risk between groups. Further large-sample studies with adequate power are warranted to verify the clinical value of low-dose alteplase for this patient population.

## 1 Introduction

Acute pulmonary thromboembolism (APTE) is a common cardiopulmonary vascular disease characterized by high incidence, high misdiagnosis rate, and high mortality. It represents a major global health problem (1). The clinical severity of acute pulmonary embolism varies widely. The short term mortality of high risk pulmonary thromboembolism (PTE) can reach 15.8–52.2% (2–5), for which thrombolytic therapy is a well-established indication.

According to the 2019 ESC/ERS guidelines on acute pulmonary embolism, patients with intermediate-high risk PTE have a significantly higher short term mortality than those with intermediate-low risk or low risk PTE. Hemodynamic deterioration and progression of right ventricular dysfunction (RVD) are the major causes of death (6). However, according to current guidelines, there are no definitive indications for thrombolysis in patients with intermediate-high risk PTE, and no specific patient subset for whom thrombolysis is unequivocally recommended. Further risk stratification is therefore required to identify patients at high risk of hemodynamic decompensation. Therefore, although current guidelines recommend anticoagulation as the initial treatment for acute intermediate-high risk PTE (7), the optimal initial strategy—anticoagulation versus thrombolysis—remains controversial.

The PEITHO trial is the largest randomized controlled trial (RCT) to date evaluating thrombolysis in intermediate-high risk PTE. The results demonstrated that the thrombolysis group (tenecteplase 0.6 mg/kg, maximum 50 mg) had a significantly lower incidence of short-term hemodynamic deterioration than the anticoagulation group. However, there were no significant differences in all-cause mortality at 7 and 30 days between the two groups. Moreover, thrombolytic therapy was associated with a substantially higher risk of major bleeding (11.5% vs. 2.4%) and hemorrhagic stroke (2.0% vs. 0.2%) (8, 9). Similar conclusions have been reported by other studies as well as multiple systematic reviews and meta-analyses (10–13).

A multicenter RCT conducted in China showed that patients receiving rt-PA at 50 mg over 2 h had a similar overall mortality compared with those receiving 100 mg over 2 h, while the reduced dose thrombolysis group exhibited a lower bleeding risk (14). This reduced dose regimen has been incorporated into the Chinese guidelines for the diagnosis and management of PTE (15). Therefore, it has become a major focus of current research whether further reduction of thrombolytic dosage in acute intermediate-high risk PTE can achieve favorable short-term and long-term outcomes without increasing major bleeding events. Zhang et al. (16) reduced the rt-PA dose to 30 mg and found that pulmonary artery systolic pressure was significantly lower in the thrombolysis group than in the anticoagulation group, with no significant difference in major bleeding rates between the two groups.

For patients with acute intermediate-high risk pulmonary embolism, identifying early predictors of hemodynamic deterioration is crucial for evaluating the benefit/risk ratio of thrombolytic therapy. Although clinical studies have reported inconsistent results regarding the association between hypoxemia at admission and prognosis in acute pulmonary embolism, some evidence suggested that the patients suffering from intermediate risk PTE with poorer outcomes had lower arterial oxygen partial pressure (17). In addition, the incidence of death or hemodynamic decompensation was significantly lower in the thrombolysis group than in the placebo group within 7 days after randomization in the PEITHO trial. It indicates that initial treatment is critical. Based on these considerations, the present study enrolled patients with acute intermediate-high risk PTE complicated by PaO_2_/FiO_2_ < 300 mmHg and applied low dose thrombolytic therapy as the initial treatment to evaluate its short-term efficacy and safety.

## 2 Methods

### 2.1 Study participants and interventions

This study was designed as a randomized, open-label, parallel-controlled clinical trial.

#### 2.1.1 Study participants

A total of 96 patients were enrolled in this study. The patients had to meet the following inclusion criteria: (1) were admitted to the Department of Respiratory and Critical Care Medicine, General Hospital of Northern Theater Command, between October 2020 and February 2025, and diagnosed acute intermediate-high risk PTE according to the 2019 ESC Guidelines for the diagnosis and management of acute pulmonary embolism developed in collaboration with the European Respiratory Society (18), with the diagnosis confirmed by computed tomography pulmonary angiography (CTPA); (2) had an arterial oxygen partial pressure to fractional inspired oxygen (PaO_2_/FiO_2_) ratio <300 mmHg; (3) were confirmed that the most recent embolic event occurred within the past 30 days according to the aggravation of symptoms during the current episode or objective evidence; (4) aged 18–75 years, regardless of sex.

Patients were excluded from the study if they met any of the following exclusion criteria: (1) had active internal bleeding or spontaneous intracranial hemorrhage within the past 6 months; (2) were operated major surgery, organ biopsy, or non-compressible vascular puncture within the past 2 weeks; (3) suffered from ischemic stroke within the past 2 months; (4) had gastrointestinal bleeding within 10 days; (5) had severe trauma within 15 days; (6) were operated neurosurgical or ophthalmologic surgery within 1 month; (7) had uncontrolled severe hypertension (systolic blood pressure >180 mmHg or diastolic blood pressure >110 mmHg); (8) underwent cardiopulmonary resuscitation; (9) had low platelet count (less than 100 × 10^9^/L); (10) were pregnancy or postpartum within 2 weeks; (11) had infective endocarditis; (12) had left atrial thrombus; (13) had aneurysm; (14) had severe hepatic or renal dysfunction; (15) had diabetic hemorrhagic retinopathy; (16) suffered from known bleeding disorders; and (17) had chronic thromboembolic pulmonary hypertension without recent acute PTE.

#### 2.1.2 Interventions

**Figure 1:**
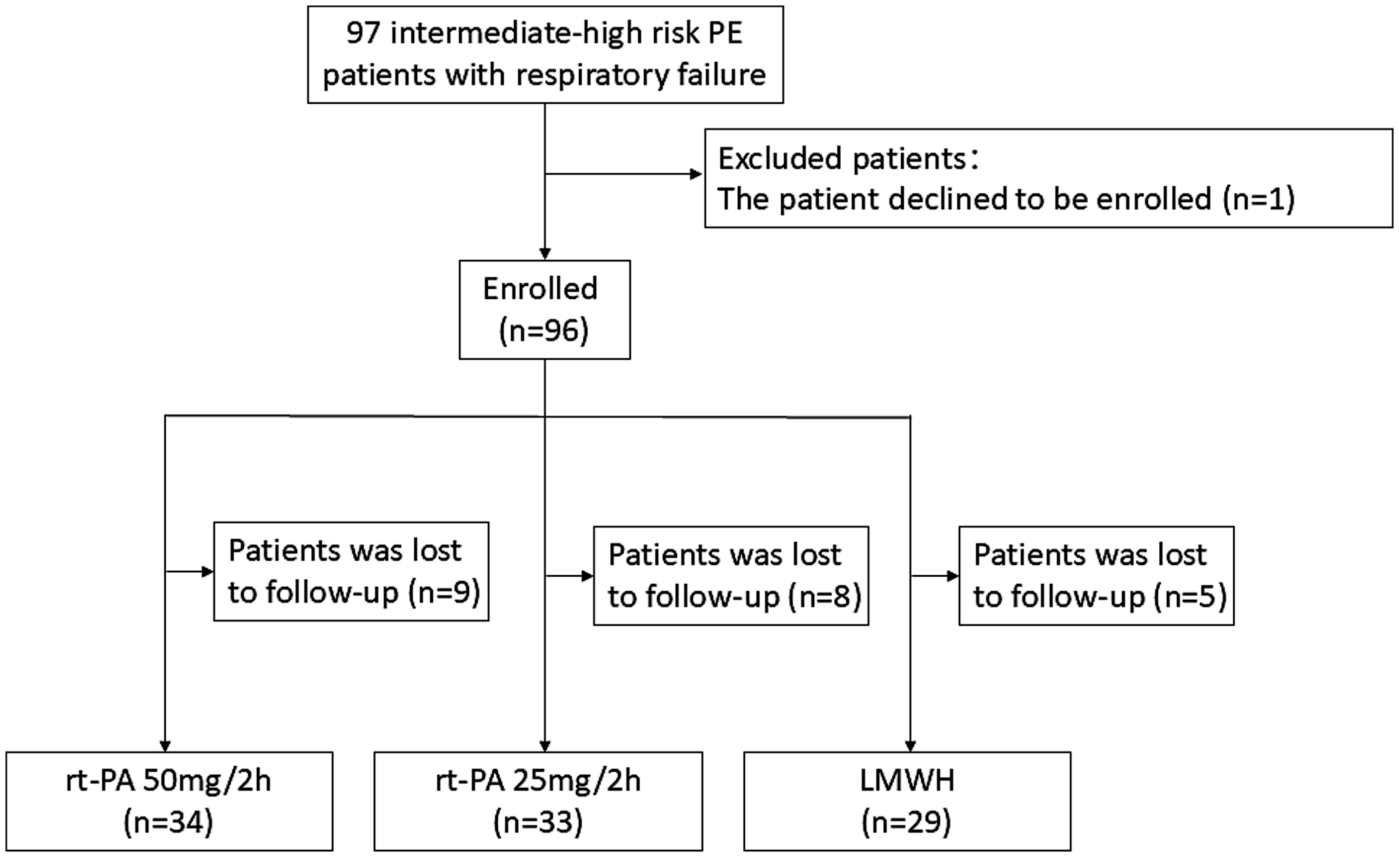
The flow chart of enrollment.

Eligible patients were randomly assigned to one of three groups (Figure 1).

(1) Conventional dose thrombolysis group: alteplase 50 mg was diluted in 50 mL sterile water and administered intravenously via syringe pump over 2 h;
(2) Low dose thrombolysis group: alteplase 25 mg was diluted in 50 mL sterile water and administered intravenously via syringe pump over 2 h;
(3) Low-molecular-weight heparin (LMWH) anticoagulation group: enoxaparin sodium (Clexane^®^, Sanofi) was administered subcutaneously at a dose of 10 mg/10 kg every 12 h.

For patients receiving thrombolytic therapy, activated partial thromboplastin time (APTT) was monitored every 4 h after completion of thrombolysis. Enoxaparin sodium was initiated when APTT returned to within twice of the normal range. Anticoagulation therapy was switched to oral agents before discharge, including warfarin, rivaroxaban, or dabigatran etexilate. During study, the initiation or intensification of thrombolysis or interventional therapy was performed according to clinical judgment if the patients in the LMWH group or low dose thrombolysis group experienced clinical deterioration or hemodynamic decompensation.

### 2.2 Data measurements

All patients maintained spontaneous breathing and received oxygen therapy via nasal cannula throughout the observation period. The fractional inspired oxygen (FiO_2_) was calculated using the standard formula: 21 + oxygen flow rate (L/min) × 4.

All patients were monitored for the PaO_2_/FiO_2_ ratio and NT-proBNP at various timepoints: before treatment (baseline), 24 h, 3 days, and 7 days after treatment. mPAP was assessed by transthoracic echocardiography at baseline, 24 h, and 7 days after treatment, while hs-TnT was measured as a baseline indicator. The primary outcome was the PaO_2_/FiO_2_ ratio. Secondary outcomes included mPAP, NT-proBNP, all-cause mortality, hemodynamic decompensation, bleeding events within 7 days of treatment, length of hospital stay, hospital costs, clinical improvement at 3 months, and the development of chronic thromboembolic pulmonary hypertension (CTEPH) or chronic thromboembolic disease (CTED) at 3 months.

### 2.3 Statistical analysis

G*Power software was used for power analysis. Under the pre-set conditions of moderate-to-large effect size (*F* = 0.35), *α* = 0.05 and 0.8 test power, the estimated sample size was 82 cases. Statistical analysis was performed using SPSS version 26.0. A mixed linear model was also used to analyze the differences in PaO_2_/FiO_2_ ratio changes and mean pulmonary artery pressure from baseline among three groups, and the analysis was performed using R 4.6.0 with lme4 package for model fitting, lmerTest for Type III ANOVA, and emmeans for post-hoc comparisons. Categorical variables were compared using the *χ^2^* test. A two-sided *p* value < 0.05 was considered statistically significant.

## 3 Results

### 3.1 Baseline clinical characteristics

A total of 96 patients were randomly assigned to three groups. There were no significant differences among the groups in age, sex, body weight, baseline vital signs (including blood pressure, heart rate, and respiratory rate), or levels of PaO_2_/FiO_2_, hs-TNT, and NT-proBNP. The proportions of comorbid hypertension, diabetes mellitus, malignancy, and chronic airway disease were also comparable across the three groups. However, the proportion of patients with deep vein thrombosis (DVT) was significantly lower in the low-molecular-weight heparin group compared with the other two groups (Table 1).

**Table 1:** Baseline clinical characteristics.

Item	rt-PA 50 mg/2 h (*n* = 34)	rt-PA 25 mg/2 h (*n* = 33)	Low-molecular-weight Heparin (*n* = 29)	*p*-value
Age (years)	61.2 ± 8.7	62.2 ± 11.8	64.9 ± 8.9	0.33
Male, *n* (%)	17 (50%)	17 (51.5%)	15 (51.7%)	0.99
Body weight (kg)	69.7 ± 9.8	69.0 ± 9.5	68.7 ± 10.8	0.92
Systolic blood pressure (mmHg)	132.9 ± 18.0	132.9 ± 18.1	135.9 ± 22.6	0.79
Diastolic blood pressure (mmHg)	82.5 ± 12.9	86.1 ± 12.0	85.0 ± 16.1	0.55
Heart rate (beats/min)	94.8 ± 17.3	95.8 ± 17.2	90.6 ± 17.3	0.46
Respiratory rate (breaths/min)	22.1 ± 3.7	23.8 ± 4.8	23.4 ± 4.3	0.23
Complicated with				
DVT, *n* (%)	24 (70.6%)	24 (72.7%)	12 (41.4%)	0.02
Hypertension, *n* (%)	14 (41.2%)	10 (30.3%)	12 (41.4%)	0.57
Diabetes mellitus, *n* (%)	6 (17.6%)	7 (21.2%)	2 (6.9%)	0.28
Malignant tumor, *n* (%)	4 (11.8%)	0	2 (6.9%)	0.14
Chronic lung disease, *n* (%)	1 (2.9%)	3 (9.1%)	3 (10.3%)	0.47
PaO_2_/FiO_2_	233.6 ± 48.2	223.3 ± 46.8	252.1 ± 34.8	0.07
hs-TNT (ng/L)	87.8 ± 173.4	57.3 ± 44.2	89.0 ± 123.3	0.52
NT-proBNP (pg/ml)	2694.2 ± 2271.6	4985.1 ± 4961.8	3459.0 ± 3338.5	0.08

### 3.2 Study outcomes

#### 3.2.1 The between-group differences in P/F ratio changes from baseline at 24 h, 3 days and 7 days post-treatment

**Figure 2:**
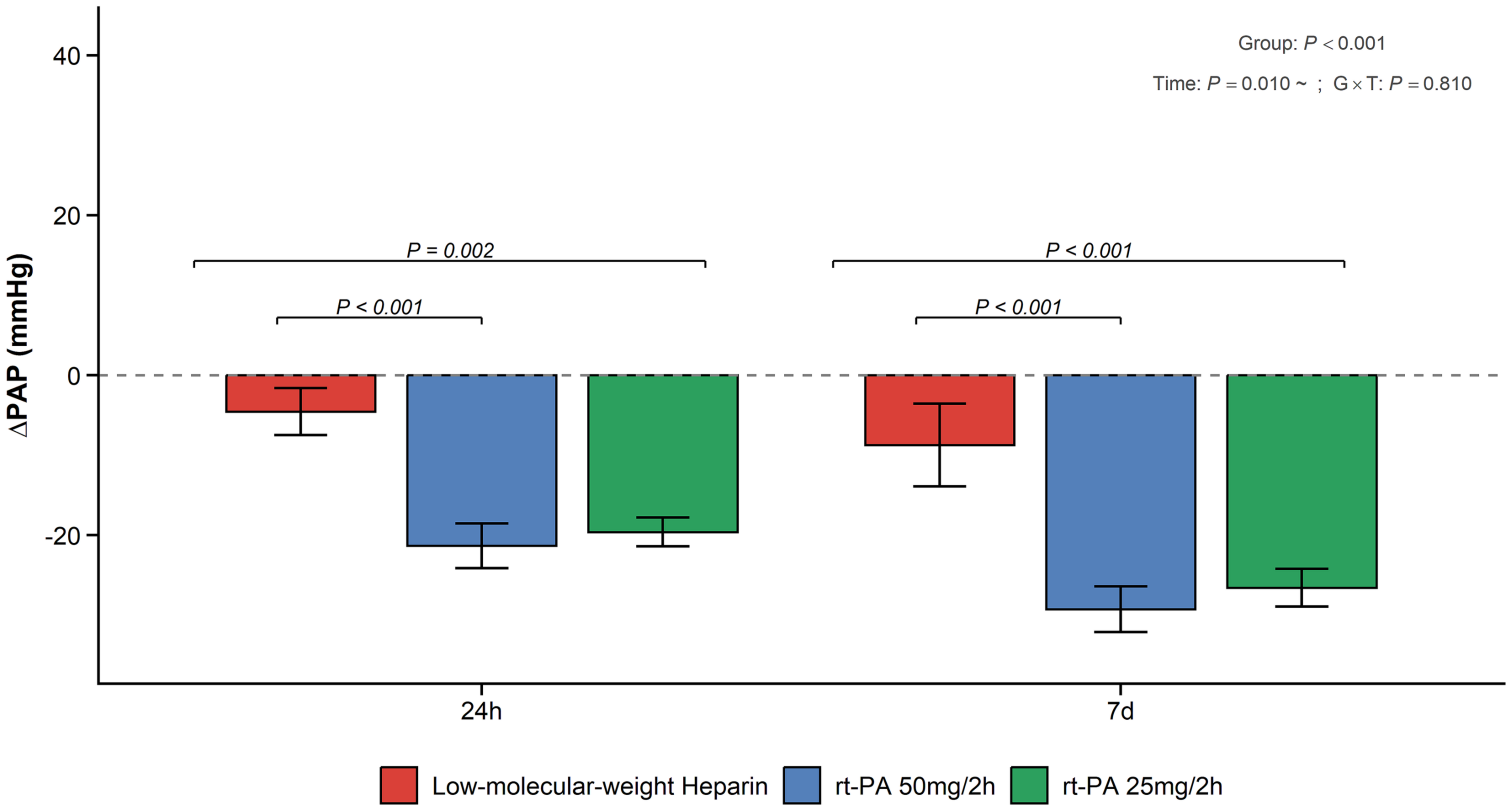
Changes in the ratio of the partial pressure of oxygen in arterial blood to the fraction of inspired oxygen (PaO_2_/FiO_2_ ratio) among the three treatment groups at 24 h, 3 days, and 7 days after treatment. The Time main effect was significant (*p* < 0.001), with an overall trend of oxygenation improvement across time. The full-dose and reduced-dose groups showed greater improvement than the anticoagulation group at 3 and 7 days, while the difference between full-dose and reduced-dose groups was not statistically significant.

The PaO_2_/FiO_2_, a key indicator for assessing the severity of respiratory failure, was the primary outcome of this study. The results demonstrated that both the low dose thrombolysis group and the conventional dose thrombolysis group showed significantly greater improvements in oxygenation index compared with the low-molecular-weight heparin anticoagulation group (*p* < 0.05) at 24 h, 3 days, and 7 days after treatment. No significant difference (*p* > 0.05) was observed between the two thrombolysis groups (Table 2, Figure 2).

**Table 2:** Changes in PaO_2_/FiO_2_ after treatment among the three groups.

Item	rt-PA 50 mg/2 h (*n* = 34)	rt-PA 25 mg/2 h (*n* = 33)	Low-molecular-weight Heparin (*n* = 29)	*p*-value
PaO_2_/FiO_2_				
24 h	42.25 ± 47.41	40.12 ± 37.34	8.48 ± 44.40	<0.01
Day 3	64.05 ± 73.99	76.47 ± 72.39	15.44 ± 51.68	<0.01
Day 7	129.7 ± 83.79	99.52 ± 99.83	40.73 ± 53.88	<0.01

#### 3.2.2 Changes in NT-proBNP at 24 hours, 3 days, and 7 days

NT-proBNP, a sensitive biomarker reflecting ventricular load and cardiac dysfunction, can directly indicate the recovery of right ventricular function in patients with acute pulmonary thromboembolism (PTE). The results showed that the reductions in NT-proBNP levels in both thrombolysis groups were significantly greater than those in the anticoagulation group at 24 h and 3 days (*p* < 0.05), There were no significant differences among the three groups on the reduction of NT-proBNP at day 7 (*p* > 0.05) (Table 3).

**Table 3:** Changes in NT-proBNP after treatment among the three groups.

Item	rt-PA 50 mg/2 h	rt-PA 25 mg/2 h	Low-molecular-weight Heparin	*p*-value
NT-proBNP				
24 h	−2,189 ± 1978	−3,071 ± 2,158	−542.1 ± 1,473	<0.01
Day 3	−2,731 ± 2,386	−2,561 ± 1,506	−912.3 ± 1,211	0.01
Day 7	−1994 ± 1,602	−3,018 ± 1939	−2095 ± 1,400	0.06

#### 3.2.3 Changes in mean pulmonary artery pressure at 24 hours and 7 days

**Figure 3:**
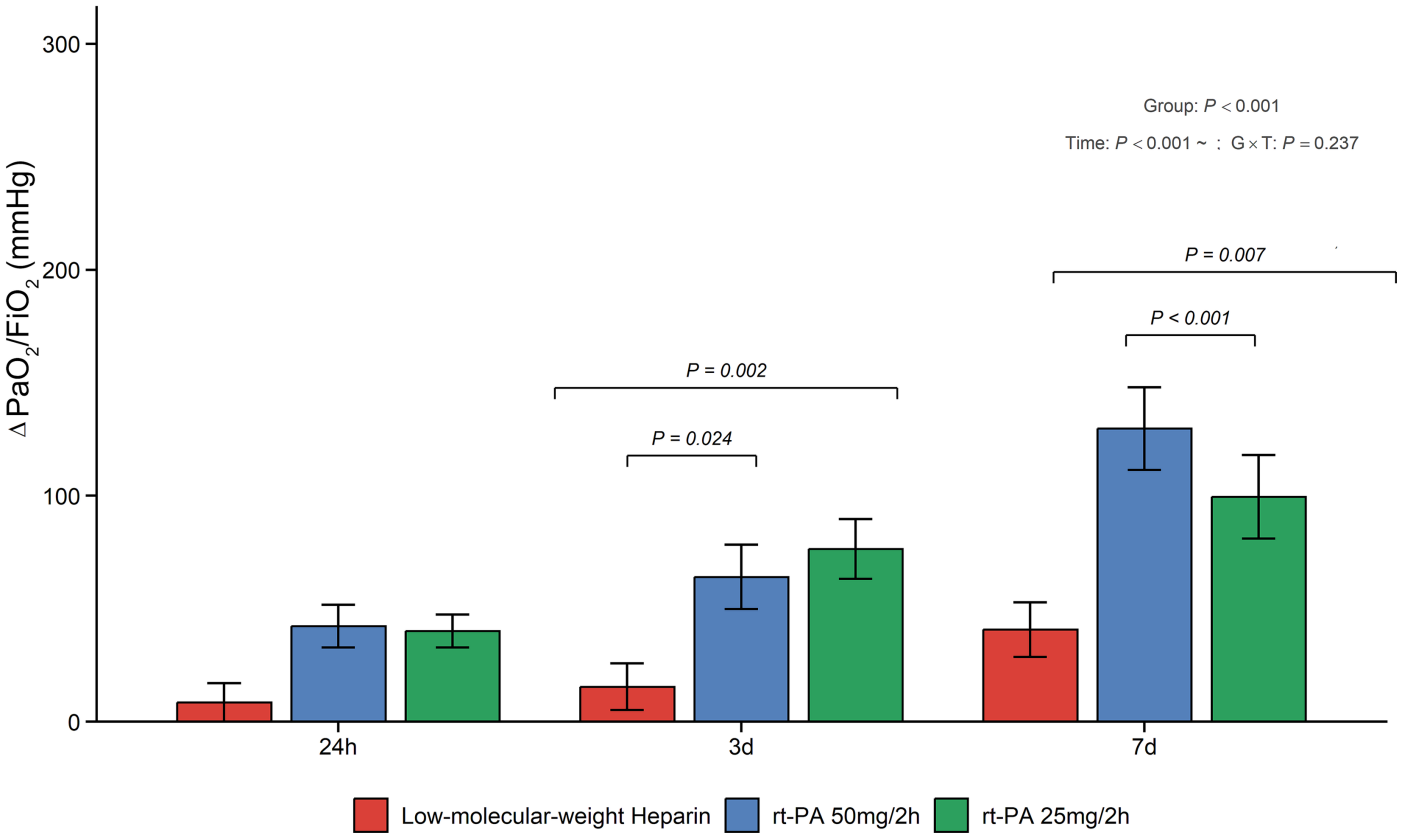
Changes in mean pulmonary artery pressure (mPAP) among the three treatment groups at 24 h and 7 days after treatment. The PAP reduction was numerically comparable between full-dose and reduced-dose groups, both demonstrating therapeutic efficacy.

Mean pulmonary artery pressure, a core parameter for evaluating the extent of pulmonary vascular obstruction and treatment response, was also assessed. The results indicated that reductions of mean pulmonary artery pressure were significantly greater in both the conventional dose group and low dose thrombolysis group compared with reduction of the anticoagulation group at both 24 h and 7 days (*p* < 0.05). No significant difference was observed between the two thrombolysis groups (*p* > 0.05) (Table 4, Figure 3).

**Table 4:** Changes in mPAP after treatment among the three groups.

Item	rt-PA 50 mg/2 h (*n* = 34)	rt-PA 25 mg/2 h (*n* = 33)	Low-molecular-weight Heparin (*n* = 29)	*p*-value
mPAP				
24 h	−21.31 ± 16.30	−19.62 ± 10.32	−4.54 ± 15.82	<0.01
Day 7	−29.26 ± 16.69	−26.56 ± 13.50	−8.71 ± 27.94	<0.01

### 3.3 Safety outcomes

One death occurred in the 96 patients within 7 days of treatment. This patient died on day 5 after receiving thrombolysis with rt-PA 50 mg. The primary cause of death was considered to be carcinomatous lymphangitis with lung cancer. One patient underwent crossover treatment but was not included in the statistical analysis of the thrombolysis groups. The patient experienced progressive clinical deterioration with hemodynamic decompensation in the anticoagulation group, and subsequently improved after he received salvage thrombolysis with rt-PA 50 mg over 2 h.

Major bleeding, as defined according to the criteria of the International Society on Thrombosis and Haemostasis, minor bleeding was defined as bleeding that did not meet the criteria for major bleeding (19). No major bleeding events were observed among the patients in this study. Among nine patients with minor bleeding events, seven cases occurred in the conventional (rt-PA 50 mg) thrombolysis group. Of these, one patient with advanced lung cancer developed hemoptysis that improved after administration of pituitrin, the other cases included two with mild hemoptysis, one with epistaxis, one with gingival bleeding, one with skin ecchymosis and gluteal hematoma, and one with increased menstruation, none of which required specific intervention. Two cases of minor bleeding occurred in the low dose (rt-PA 25 mg) thrombolysis group, including one case of blood streaked sputum and one case of epistaxis which improved with local management (Table 5).

**Table 5:** Safety outcome events during the 7 days.

Item	rt-PA 50 mg/2 h (*n* = 34)	rt-PA 25 mg/2 h (*n* = 33)	Low-molecular-weight Heparin (*n* = 29)	*p*-value
Death	1	0	0	0.40
Hemodynamic decompensation	0	0	1	0.31
Major bleeding	0	0	0	NA
Minor bleeding	7	2	0	0.01

### 3.4 Clinical outcomes

There were no significant differences among the three groups in total length of hospital stay, ICU stay, and treatment costs according to clinical outcomes within 7 days. No statistically significant differences were observed among the groups in terms of symptom improvement or progression to CTED or CTEPH at 3-month follow-up (*p* > 0.05) (Table 6).

**Table 6:** Clinical outcome during follow-up.

t	rt-PA 50 mg/2 h	rt-PA 25 mg/2 h	Low-molecular-weight Heparin	*p*-value
Total hospital stays (days)	10.4 ± 3.5	11.6 ± 4.5	10.4 ± 2.4	0.31
ICU stays (days)	3.3 ± 1.3	3.2 ± 1.2	3.4 ± 1.6	0.92
Hospital cost (RMB)	20734.8 ± 7157.3	20973.5 ± 7369.2	22274.2 ± 9567.3	0.73
Improvement rate at 3-month follow-up	20/25	24/25	21/24	0.22
Proportion of progression to CTEPH or CTED	4/25	3/25	9/24	0.07

## 4 Discussion

Thrombolytic therapy is recommended as rescue treatment for high risk PTE patients with hemodynamic decompensation according to international guidelines (7, 20). However, the optimal therapeutic strategy for patients with acute intermediate-high risk PTE who are hemodynamically stable but present with right ventricular dysfunction and elevated cardiac biomarkers remains a major focus of clinical research. Beyond systemic thrombolysis and anticoagulation, catheter-directed therapies and suction embolectomy have also emerged as alternative reperfusion strategies for this controversial risk subgroup, though their clinical application and efficacy compared with conventional approaches require further validation. In particular, dose optimization as a key issue has been highlighted on balancing therapeutic efficacy against bleeding risk.

A meta-analysis demonstrated that initial thrombolytic therapy for acute intermediate-risk PTE was associated with a trend toward reduced mortality, decreased risk of clinical deterioration, lower recurrence rates, and improved right ventricular function with reduced pulmonary artery pressure, but significantly increased bleeding risk compared with anticoagulation alone (21). Another systematic review and meta-analysis compared the efficacy and safety of standard-dose thrombolysis (rt-PA 100 mg), reduced-dose thrombolysis (rt-PA ≤ 50 mg or 0.6 mg/kg), and anticoagulation alone in acute PTE (22). The results showed no significant differences in all-cause mortality or PTE recurrence among the three groups, whereas bleeding complications displayed a clear dose-dependent pattern. Low-dose rt-PA significantly reduced bleeding risk compared with standard-dose rt-PA, and was associated with only a higher incidence of minor bleeding relative to anticoagulation alone. As reported by Song et al., low-dose alteplase (≤50 mg) significantly decreased short-term mortality and showed a superior safety profile compared with high-dose alteplase (>50 mg) in patients with intermediate-risk pulmonary embolism, achieving a more optimal efficacy–risk balance (23). These findings support the potential feasibility of low-dose thrombolysis as an initial therapeutic strategy for acute intermediate-high risk PTE.

Nevertheless, not all patients with acute intermediate-high risk PTE require thrombolytic therapy. It is critical to identify the patients at high risk of deterioration. Compared with approaches relying solely on clinical characteristics to identify patients who may benefit from thrombolysis (24, 25), the prognostic models that integrate clinical features, biochemical markers, and imaging evidence of right ventricular dysfunction demonstrate superior predictive performance (18), such as the Bova score and TELOS score (26, 27). However, these models primarily emphasize cardiac related parameters, while oxygenation indices are rarely incorporated. Nearly 60% of PTE patients present with hypoxemia according to previous reports (28), and respiratory dysfunction is also an important contributor to mortality. However, there have been no published studies addressing whether thrombolysis or anticoagulation in patients with acute intermediate-high risk PTE complicated by PaO_2_/FiO_2_ ratio < 300 mmHg is preferable as initial therapy to date, and what the optimal thrombolytic dose should be. The present study helps to fill this knowledge gap.

Our results suggested that intravenous administration of 25 mg rt-PA over 2 h may be both safe and effective in patients with acute intermediate-high risk PTE and PaO_2_/FiO_2_ ratio < 300 mmHg. Low-dose thrombolysis was associated with significant early improvements in oxygenation, reductions in pulmonary artery systolic pressure, and recovery of right ventricular function. These findings are consistent with the limited number of published studies in this field. In the MOPETT trial (29), the thrombolysis group (maximum rt-PA dose ≤ 50 mg) showed a significant reduction in pulmonary artery systolic pressure within 48 h compared with the anticoagulation group, and this effect was sustained during a mean follow-up of 28.5 months. Similarly, the patients treated with thrombolysis (rt-PA 30 mg) experienced a significantly greater reduction in pulmonary artery systolic pressure within 24 h compared with those receiving anticoagulation alone (17.0 ± 10.2 mmHg vs. 4.6 ± 9.8 mmHg, *p* < 0.001), along with improved right ventricular function in another small-sample study (16).

The mechanisms of hypoxemia in acute pulmonary embolism are primarily related to ventilation/ perfusion mismatch and decreased cardiac output (30–32). Consequently, acute PTE may substantially impair oxygenation even in the presence of preserved systemic blood pressure. Previous studies had shown that hypoxemia frequently coexists with right ventricular dysfunction (33, 34). Becattini et al. reported that the intermediate-high risk patients with arterial oxygen saturation (SaO_2_) < 88% had a significantly higher 30-day mortality than those with the SaO_2_ ≥ 88% (21.7% vs. 5.8%). And the SaO_2_ < 88% on inhaling room air was an independent predictor of mortality (35). Admission hypoxemia assessed by PaO_2_/FiO_2_ has been associated with an increased risk of early hemodynamic deterioration and demonstrated superior predictive value compared with baseline heart rate or echocardiographic parameters in patients with intermediate risk acute PTE (36).

In the present study, improvement in the PaO_2_/FiO_2_ ratio in the anticoagulation-only group was significantly slower than that in the thrombolysis groups. This suggests that thrombolytic therapy may be more time-sensitive than anticoagulation alone in intermediate-high risk patients with a PaO_2_/FiO_2_ ratio < 300 mmHg. These findings indicate that the PaO_2_/FiO_2_ ratio could be used as an important stratification parameter when considering thrombolysis in acute intermediate-high risk PTE. For patients with a PaO_2_/FiO_2_ ratio < 300 mmHg, low-dose thrombolysis may be a reasonable option. This “oxygenation-centered dynamic stratification strategy” might help to further optimize individualized treatment for acute intermediate-high risk PTE.

Bleeding complication remains the principal limitation to the widespread use of thrombolysis in acute intermediate-high risk PTE. Safety outcomes from the present study showed that no major bleeding events were observed within 7 days in any of the three groups. Moreover, the incidence of minor bleeding was numerically lower in the low-dose thrombolysis group than in the conventional-dose group. Notably, one patient in the anticoagulation-only group required rescue thrombolysis due to clinical deterioration, whereas no such cases were documented in the low-dose thrombolysis group, Owing to insufficient statistical power, we cannot draw definitive conclusions about intergroup differences in bleeding risk. These findings suggest that low-dose thrombolysis may present a favourable safety trend, and might potentially reduce the need for secondary interventions following anticoagulation failure as well as adverse events related to disease progression. These results are in line with those reported by Zhang et al. (16). In addition, thrombolytic therapy in this study did not appear to prolong ICU stay, hospital stay, or increase the overall economic burden compared with anticoagulation alone. Given the limited statistical power, low-dose thrombolysis may hold meaningful clinical value by potentially maintaining therapeutic efficacy while showing a trend toward reduced treatment-related risks in acute intermediate-high risk PTE.

This study has several limitations that should be acknowledged. First, it was a single-center study with a relatively small sample size, which may have introduced selection bias and compromised statistical power. Therefore, multicenter studies with larger sample sizes are warranted to further validate the present findings. Second, the inclusion criteria were relatively restrictive, limited to patients aged 18–75 years, while excluding individuals with severe comorbidities, pregnant women, and other special populations. This may restrict the generalizability of the conclusions to a broader patient cohort. In addition, the follow-up duration was relatively short: the study focused primarily on short-term outcomes within 7 days, with only 3 months of follow-up completed, thus lacking long-term prognostic data. Although low-dose thrombolysis demonstrated favorable short-term efficacy and safety profiles, extended follow-up is required to verify its long-term thrombolytic efficacy, ability to prevent recurrence, and potential late adverse events.

## 5 Conclusion

For hemodynamically stable patients with acute intermediate-high risk PTE and a PaO_2_/FiO_2_ ratio < 300 mmHg, intravenous low-dose rt-PA (25 mg infused over 2 h) yields more rapid early oxygenation improvement, superior reduction in pulmonary artery pressure, and earlier recovery of right ventricular function relative to anticoagulation alone. This low-dose regimen exhibits a favorable safety profile, with a lower risk of bleeding and fewer cases requiring rescue thrombolysis. The PaO_2_/FiO_2_ ratio represents a useful risk-stratification biomarker for identifying patients eligible for low-dose thrombolysis, and this oxygenation-centered stratification strategy supports personalized therapeutic management for this specific subgroup. However, limitations including the single-center design, small sample size, and short follow-up period warrant large multicenter trials with prolonged observation to validate the sustained efficacy and long-term safety of low-dose thrombolysis in this cohort.

## Data Availability

The original contributions presented in the study are included in the article/supplementary material, further inquiries can be directed to the corresponding authors.
